# Retraction: Kallikrein Gene-Modified EPCs Induce Angiogenesis in Rats with Ischemic Hindlimb and Correlate with Integrin αvβ3 Expression

**DOI:** 10.1371/journal.pone.0353531

**Published:** 2026-07-13

**Authors:** The PLOS One Editors

After this article [1,2] was published, concerns were raised regarding results presented in Figs 5, 9, and 10. Specifically:

The following panels appear to partially overlap:

- Fig 5A Ad/GFP-EPCs, Fig 5A Ad/hTK-EPCs, Fig 9A EPCs PBS, and Fig 9A Ad/*hTK*-EPCs cyclo.
- Fig 10A Ad/*hTK*-EPCs cyclo and Fig 10A EPCs PBS.

The corresponding author stated that the Fig 5A Ad/GFP-EPCs, Fig 5A Ad/hTK-EPCs, Fig 9A EPCs PBS, and Fig 10A Ad/*hTK*-EPCs cyclo panels are incorrect. They provided replacement images for these panels and the available data underlying the article. Upon editorial review, PLOS identified additional concerns for the underlying image data provided that call into question the reliability of the reported results.

In light of the above unresolved concerns, the *PLOS One* Editors retract this article.

QDL did not agree with the retraction. SSF, FJL, YYW, ABY, YLQ, XM, JRL, BCL, and YZ either did not respond directly or could not be reached.
